# Immunoglobulin E and Mast Cell Proteases Are Potential Risk Factors of Human Pre-Diabetes and Diabetes Mellitus

**DOI:** 10.1371/journal.pone.0028962

**Published:** 2011-12-16

**Authors:** Zhen Wang, Hong Zhang, Xu-Hui Shen, Kui-Li Jin, Guo-fen Ye, Li Qian, Bo Li, Yong-Hong Zhang, Guo-Ping Shi

**Affiliations:** 1 Department of Clinical Medicine, School of Medicine, Huzhou Teachers College, Huzhou, Zhejiang, China; 2 Department of Nursing Medicine, School of Medicine, Huzhou Teachers College, Huzhou, Zhejiang, China; 3 Huzhou Center for Disease Prevention and Control, Huzhou, Zhejiang, China; 4 Aishan Street Community Health Center, Huzhou, Zhejiang, China; 5 Longquan Street Community Health Center, Huzhou, Zhejiang, China; 6 Huanzhu Rural Hospital, Huzhou, Zhejiang, China; 7 Department of Epidemiology, Soochow University School of Radiation Medicine and Public Health, Suzhou, Jiangsu, China; 8 Department of Medicine, Brigham and Women's Hospital and Harvard Medical School, Boston, Massachusetts, United States of America; Brigham & Women's Hospital, and Harvard Medical School, United States of America

## Abstract

**Background:**

Recent studies have suggested that mast-cell activation and inflammation are important in obesity and diabetes. Plasma levels of mast cell proteases and the mast cell activator immunoglobulin E (IgE) may serve as novel inflammatory markers that associate with the risk of pre-diabetes and diabetes mellitus.

**Methods and Results:**

A total of 340 subjects 55 to 75 years of age were grouped according to the American Diabetes Association 2003 criteria of normal glucose tolerance, pre-diabetes, and diabetes mellitus. The Kruskal-Wallis test demonstrated significant differences in plasma IgE levels (*P* = 0.008) among groups with different glucose tolerance status. Linear regression analysis revealed significant correlations between plasma levels of chymase (*P* = 0.030) or IgE (*P* = 0.022) and diabetes mellitus. Ordinal logistic regression analysis showed that IgE was a significant risk factor of pre-diabetes and diabetes mellitus (odds ratio [OR]: 1.674, *P* = 0.034). After adjustment for common diabetes risk factors, including age, sex, hypertension, body-mass index, cholesterol, homeostatic model assessment (HOMA) index, high-sensitivity C-reactive protein (hs-CRP), and mast cell chymase and tryptase, IgE remained a significant risk factor (OR: 1.866, *P* = 0.015). Two-variable ordinal logistic analysis indicated that interactions between hs-CRP and IgE, or between IgE and chymase, increased further the risks of developing pre-diabetes and diabetes mellitus before (OR: 2.204, *P* = 0.044; OR: 2.479, *P* = 0.033) and after (OR: 2.251, *P* = 0.040; OR: 2.594, *P* = 0.026) adjustment for common diabetes risk factors.

**Conclusions:**

Both IgE and chymase associate with diabetes status. While IgE and hs-CRP are individual risk factors of pre-diabetes and diabetes mellitus, interactions of IgE with hs-CRP or with chymase further increased the risk of pre-diabetes and diabetes mellitus.

## Introduction

The development of diabetes mellitus — and in particular, of type 2 diabetes mellitus (type 2 DM) — involves multiple stages and is complicated by multiple factors, such as obesity and cardiovascular disease. The exact mechanisms leading to diabetes mellitus, however, remain unknown. Pre-diabetes refers to the intermediate states between normal glucose tolerance (NGT) and type 2 DM and is the precursor of type 2 DM. Individuals with pre-diabetes often bear clusters of cardiovascular disease risk factors [1]. In recent years, studies have focused on the inflammatory risk factors of pre-diabetes and type 2 DM [1]–[6]. Elevated plasma levels of inflammatory high-sensitivity C-reactive protein (hs-CRP) were seen in two Asian-population–based cohorts of pre-diabetes [7]. Mast cells are essential components of asthma and allergic responses [8], [9], but recent studies have shown that these cells are important in diet-induced obesity and type 2 DM. Mice lacking mast cells or receiving the mast cell inhibitors cromolyn or ketotifen (Zaditor) are fully protected from developing type 2 DM [10]. By releasing the mast-cell–specific serine proteases chymase [11] and trypase [12], these “allergy cells” contribute to neovascularization and vascular-cell apoptosis.

One of the best-known mechanisms of mast cell activation is the binding of immunoglobulin E (IgE) to its high-affinity receptor FcεR1 on the mast cell surface. After IgE binding, mast cells release histamine, mast cell protease, proteoglycan, cytokines, and chemokines [13], [14]. Many of these inflammatory mediators associate with diabetes mellitus [15]–[17]. Plasma levels of mast cell chymase and tryptase are greatly elevated in patients with acute myocardial infarction (AMI) or unstable angina pectoris (UAP) [18]. We recently showed that human plasma IgE levels also associate with coronary artery intima thickness and rupture. IgE levels in patients with AMI were double those in subjects with stable angina pectoris (SAP) or without coronary heart disease (CHD) [19]. In human atherosclerotic lesions, IgE and its receptor FcεR1 are localized to macrophage-rich areas, as well as to smooth-muscle cells (SMCs) and endothelial cells (ECs). In atherosclerosis-prone apolipoprotein E–deficient (*Apoe^−/−^*) mice, the absence of FcεR1 α-subunit reduced atherosclerotic lesion sizes by 75% in the thoracic to abdominal aorta, and by more than 50% in the aortic arch. Mechanistically, we demonstrated that IgE activated macrophage, EC, and SMC signaling transduction, apoptosis, and cytokine and chemokine production [19] — all of which participate in metabolic diseases [10], [20], [21]. The current study is designed to examine whether IgE and mast cell proteases associate with infammation and diabetes status in a Chinese population from a pre-diabetes study.

## Materials and Methods

### Study population

This study is part of the Pre-diabetes Intervention Project (PDIP), which began in 2008 at the School of Medicine, Huzhou Teacher's College, Zhejiang, China. From July to August 2008, a total of 3163 volunteers 55 to 75 years of age, from three neighborhood communities in the city of Huzhou, were invited for pre-diabetes screening. After excluding subjects with known DM or cardiovascular disease, cerebrovascular disease, malignant disease, chronic liver disease, or kidney failure, and those taking medications, 1500 volunteers were invited for a fasting glucose test and a 2-hour oral glucose tolerance test (2h-OGTT) as part of the pre-diabetes screening. Of those, 1197 accepted the invitation and participated in both tests from September to December 2008. Among the subjects, 807 (67.42%) had normal glucose tolerance (NGT), 267 (22.30%) were diagnosed with pre-diabetes, and 123 (10.28%) were diagnosed with DM. One year after the initial visit, 267 pre-diabetes subjects, 123 DM subjects, and 100 randomly-selected NGT subjects were invited for anthropometric measurements and clinical tests. A total of 340 subjects participated in the final study, and among them, 71 had normal glucose tolerance (NGT), 189 had pre-diabetes, and 80 had DM. This study was approved by the Huzhou City Ethics Committee, and all subjects gave written, informed consent prior to participating.

### Data collection

Demographic data, including age and sex, were collected at the registry. Anthropometric measurements (body height and weight, waist and hip circumference) were performed with the subjects wearing light underwear and without shoes. The biochemical parameters were measured in the Clinical Biochemistry Unit of Huzhou First Hospital, a teaching hospital of the School of Medicine. All participants were fasted for 10–12 hours to determine plasma glucose levels, lipid profiles, and insulin levels. To perform 2h-OGTT, participants were asked not to smoke or to partake in vigorous exercise during 2 hours of sample collection, and rested for a minimum of 15 minutes. An antecubital blood sample was drawn immediately before and 2 hours after glucose ingestion. The glucose oxidase method was used (Japanese OLYMPUS AV640 Automatic Biochemical Analyzer) to measure plasma glucose concentration. Plasma hs-CRP levels were determined by a high-sensitivity latex-enhanced nephelometric assay (Sysmex Wuxi Co., Ltd., Wuxi, China). Plasma insulin (Abbott Japan Co., Ltd., Tokyo, Japan) and IgE (DiaSys Diagnostic Systems GmbH, Holzheim, Germany) levels were measured using a radioimmunoassay. Plasma total cholesterol (TC), triglyceride (TG), low-density lipoprotein cholesterol (LDL-C), and high-density lipoprotein cholesterol (HDL-C) were determined by an enzymatic assay (Beijing Leadman Biochemistry Co., Ltd., Beijing, China). Plasma chymase and tryptase levels were determined as described previously [18].

### Clinical criteria

Subjects with diabetes and pre-diabetes were grouped according to American Diabetes Association 2003 criteria [22]. Diabetes was classified with a fasting plasma glucose (FPG) ≥7.0 mmol/L or 2h-OGTT ≥11.1 mmol/L, or receiving hypoglycemic medication; whereas pre-diabetes was defined as FPG ≥5.6 and <7.0 mmol/L or 2h-OGTT ≥7.8 and <11.1 mmol/L. Subjects were classified as having a normal glucose profile if FPG <5.6 mmol/L and 2h-OGTT <7.8 mmol/L.

Based on China 2006 Blood Pressure Control Criteria and China Prevention and Treatment Classification Recommendation on Dyslipidemia [23], hypertension was defined as systolic blood pressure (SBP) and/or diastolic blood pressure (DBP) ≥140/90 mmHg, or as receiving blood-pressure–lowering medications; high TG was defined as a fasting plasma TG ≥1.70 mmol/L, low HDL-C as a fasting HDL-C ≤0.9 mmol/L, high TC as TC ≥5.72 mmol/L, and low LDL-C as a fasting LDL-C ≤3.64 mmol/L. Based on the China Obesity Task Group Recommendation [24], general obesity was classified as body-mass index (BMI) ≥24 kg/m^2^; central obesity was defined as a waist circumference (WC) ≥80 cm in females or 85 cm in males. High waist-to-hip ratio (WHR) was classified as normal/abnormal according to upper quartile (P_75_ = 0.93). Homeostatic model assessment–insulin resistance (HOMA-IR = value of FPG × value of fasting insulin/22.5) was classified as normal/abnormal according to upper quartile (P_75_ = 2.58), and HOMA-β cell function [HOMA-β = 20× value of fasting insulin/(FPG-3.5)] was classified as normal/abnormal according to bottom quartile (P_25_ = 38.17). Hyperinsulinemia was classified as normal/abnormal according to upper quartile (P_75_ = 9.38 mIU/L).

### Statistical analysis

We used the mean and standard deviation of continuous and normal distributional variables and the median and interquartile range of continuous but skewed distributional variables. Data were analyzed using one-way analysis of variance (ANOVA), chi-square test, linear regression test, Kruskal-Wallis test, ordinal logistic model, or binary logistic model. All statistical analyses were conducted using SPSS statistical software (version 11.0).

## Results

### Population distribution

Basic characteristics of all 340 participants are shown in Table 1. All data with normal distributions were expressed as mean ± SD (standard deviation); otherwise, data were expressed as median and interquartile ranges. Sex was expressed as the percentage of males, and plasma tryptase levels were expressed as percentages of those among the upper quartile (>2.74 ng/mL). Of the 340 participants — 96 men (average age, 67.84±5.35 years) and 244 women (average age, 64.30±6.02 years) — 71 (20.9%) were classified as NGT subjects, 189 (55.6%) as pre-diabetes subjects, and 80 (23.6%) as diabetes subjects. One-way ANOVA, Kruskal-Wallis test, or chi-square test demonstrated that FPG, 2h-OGTT, HOMA-β, HOMA-IR, TG, WC, AC, WHR, hs-CRP, and IgE levels were significantly higher in patients with pre-diabetes or diabetes mellitus than in the normal glucose group (NGG). Compared with patients in the NGG, levels of FPG, 2h-OGTT, fasting insulin, HOMA-β, HOMA-IR, SBP, WHR, hs-CRP, and IgE were significantly higher in the pre-diabetes group (PDG); levels of FPG, 2h-OGTT, fasting insulin, HOMA-β, HOMA-IR, TC, TG, BMI, WC, AC, WHR, hs-CRP, IgE, and chymase also were significantly higher in the diabetes mellitus group (DMG) (Table 1). Linear regression analysis revealed significant correlations in levels of FPG, 2h-OGTT, fasting insulin, HOMA-β, HOMA-IR, TC, TG, HDL-C, BMI, WC, AC, WHR, IgE, and chymase among the NGG, PDG, and DMG (Table 1).

**Table 1:** Biochemical and anthropometric parameters in 340 subjects with different glucose status, grouped according to fasting and post-load glucose levels.

Variable	NGG (n = 71)	PDG (n = 189)	DMG (n = 80)	*P* value^d^	*P* value^e^
Age (years)^a^	65.13±6.17	65.49±6.20	65.00±5.23	0.875	0.875
Sex (%, male)^b^	21.1	31.7	26.3	0.533	0.533
FPG (mmol/L)^c^	4.98 (4.60–5.19)	5.76 (5.53–6.04)†	6.73 (6.24–7.58)‡	<0.001	<0.001
2h-OGTT (mmol/L)^a^	5.50±1.25	7.27±1.29†	13.44±1.33‡	<0.001	<0.001
Fasting insulin (mU/L)^a^	5.42±1.82	6.41±1.70†	6.50±1.75†	0.169	0.049
HOMA-β^a^	88.13±2.54	62.51±2.42†	42.47±3.05‡	<0.001	<0.001
HOMA-IR^c^	1.14 (0.75–1.88)	1.65 (1.20–2.34)†	1.86 (1.41–2.91)‡	<0.001	<0.001
SBP (mmHg)^a^	134.00±15.89	139.37±17.54†	139.87±21.93	0.080	0.057
DBP (mmHg)^a^	77.93±7.41	79.03±9.40	78.78±10.80	0.7000	0.595
TC (mmol/L)^a^	4.87±0.97	5.04±1.05	5.20±0.96†	0.136	0.046
TG (mmol/L)^c^	1.22 (0.79–1.79)	1.46 (1.01–1.98)	1.63 (1.20–2.36)†	0.005	0.037
HDL-C (mmol/L)^c^	1.23 (1.06–1.42)	1.20 (1.05–1.37)	1.16 (0.99–1.35)	0.257	0.030
LDL-C (mmol/L)^c^	2.43 (2.04–3.00)	2.47 (2.11–2.98)	2.58 (2.18–3.00)	0.455	0.234
BMI (kg/m^2^)^a^	23.52±3.39	24.29±3.38	24.76±3.14†	0.073	0.024
WC (cm)^a^	79.88±8.61	83.52±8.85†	83.53±10.93†	0.015	0.021
AC (cm)^a^	90.79±7.58	92.74±7.46	93.94±6.81†	0.033	0.010
WHR^c^	0.87 (0.83–0.90)	0.89 (0.86–0.93)†	0.90 (0.87–0.95)†	<0.001	0.002
hs-CRP (mg/dL)^c^	2.00 (1.00–3.00)	5.00 (3.00–8.80)†	5.00 (3.00–8.00)†	<0.001	0.475
IgE (IU/L)^c^	9.00 (4.00–26.00)	16.00 (5.00–55.50)†	25.00 (7.00–72.50)†	0.008	0.022
Chymase (ug/ml)^c^	18.17 (12.37–25.28)	20.91 (13.30–26.61)	23.05 (14.52–26.61)†	0.083	0.030
Tryptase (%, >2.74 ng/ml)^b^	22.1	24.1	30.4	0.573	0.208

NGG: normal glucose group; PDG: pre-diabetes group; DMG: diabetes mellitus group; FPG: fasting plasma glucose; 2h-OGTT: 2-hour oral glucose tolerance test; BMI: body-mass index; SBP: systolic blood pressure; DBP: diastolic blood pressure; WC: waist circumference; AC: abdominal circumference; WHR: waist-to-hip ratio; TC: total cholesterol; TG: triglyceride; HDL-C: high-density lipoprotein cholesterol; LDL-C: low-density lipoprotein cholesterol; HOMA: homeostatic model assessment; IgE: immunoglobulin E; hs-CRP: high-sensitivity C-reactive protein.

a ). Variable is described in mean and standard deviation (data with normal distribution); one-way ANOVA.

b ). Chi-square test.

c ). Variable is described in median and interquartile range (data with skewed distribution); non-parametric Kruskal-Wallis test.

d ). Combined P values with corresponding methods in a–c.

e ). Linear regression analysis.

† *P*<0.05, compared with NGG;

‡ *P*<0.01, compared with NGG.

### Plasma IgE, chymase, and tryptase as independent risk factors of pre-diabetes and diabetes mellitus

Ordinal logistic regression analysis examined whether IgE and mast cell protease chymase and tryptase are significant risk factors for pre-diabetes and diabetes mellitus in this Chinese population, compared with variables from the NGG. Four different models of adjustment were used, based on selections of similar variables: Models one and three used HOMA-IR, whereas models two and four used HOMA-β. Models one and two used WC as the obesity standard, whereas models three and four used BMI (Table 2). Before adjustment for any of the variables listed in Table 2, hypertension, WC, WHR, HOMA-IR, HOMA-β, hs-CRP, and IgE were significant risk factors for pre-diabetes and diabetes mellitus. After adjustment in all four models, however, only hypertension and HOMA-IR (or HOMA-β) remained significant risk factors for pre-diabetes and diabetes mellitus. Among the remaining variables, IgE remained a significant pre-diabetes and diabetes mellitus risk factor in three models of adjustment. In contrast, mast cell chymase and tryptase were not significant risk factors for pre-diabetes and diabetes mellitus before or after adjustment for all common diabetes variables (Table 2).

**Table 2:** Influence of different variables on the risk of developing pre-diabetes and diabetes mellitus — ordinal logistic model.

Variable	Before adjustment		After adjustment (Model one)		After adjustment (Model two)		After adjustment (Model three)		After adjustment (Model four)	
	OR (95.0% CI)	Sig	OR (95.0% CI)	Sig	OR (95.0% CI)	Sig	OR (95.0% CI)	Sig	OR (95.0% CI)	Sig
Age	0.981 (0.644–1.495)	0.929	0.752 (0.473–1.196)	0.228	0.791 (0.494–1.269)	0.331	0.786 (0.498–1.243)	0.303	0.821 (0.516–1.305)	0.403
Sex	0.872 (0.553–1.375)	0.556	0.792 (0.481–1.304)	0.359	1.056 (0.633–1.762)	0.835	0.814 (0.498–1.333)	0.414	1.094 (0.662–1.810)	0.725
Hypertension	1.952 (1.278–2.980)	0.002	1.741 (1.105–2.743)	0.017	1.930 (1.214–3.068)	0.005	1.725 (1.102–2.699)	0.017	1.848 (1.172–2.914)	0.008
WC	1.554 (1.012–2.384)	0.044	1.219 (0.761–1.954)	0.410	2.347 (1.433–3.845)	0.001	-	-	-	-
WHR	2.513 (1.519–4.158)	<0.001	-	-	-	-	-	-	-	-
BMI	1.477 (0.974–2.241)	0.067	-	-	-	-	1.062 (0.671–1.680)	0.797	1.966 (1.231–3.140)	0.005
TC	1.398 (0.855–2.284)	0.182	1.304 (0.766–2.219)	0.327	1.237 (0.721–2.123)	0.440	1.323 (0.775–2.260)	0.305	1.292 (0.752–2.220)	0.354
TG	1.514 (0.991–2.314)	0.055	1.101 (0.685–1.769)	0.691	1.515 (0.931–2.465)	0.094	1.095 (0.685–1.752)	0.704	1.549 (0.956–2.510)	0.076
Lower HDL-C	1.026 (0.492–2.138)	0.945	-	-	-	-	-	-	-	-
Higher LDL-C	1.294 (0.492–3.403)	0.602	-	-	-	-	-	-	-	-
Hyperinsulinemia	1.179 (0.734–1.893)	0.497	-	-	-	-	-	-	-	-
HOMA-β index	3.574 (2.177–5.867)	<0.001	-	-	6.445 (3.567–11.643)	<0.001	-	-	6.035 (3.410–10.682)	<0.001
HOMA-IR index	2.317 (1.323–3.452)	0.002	2.039 (1.179–3.524)	0.011	-	-	2.113 (1.223–3.650)	0.007	-	-
Hs-CRP	1.030 (1.052–2.686)	0.030	1.446 (0.871–2.403)	0.154	1.222 (0.729–2.050)	0.447	1.443 (0.879–2.368)	0.147	1.290 (0.779–2.134)	0.322
IgE	1.674 (1.039–2.698)	0.034	1.713 (1.031–2.846)	0.038	1.599 (0.955–2.679)	0.074	1.866 (1.128–3.087)	0.015	1.763 (1.058–2.937)	0.030
Chymase	1.387 (0.858–2.244)	0.182	1.214 (0.730–2.020)	0.241	1.431 (0.850–2.410)	0.178	1.122 (0.677–1.859)	0.655	1.218 (0.730–2.033)	0.449
Tryptase	1.283 (0.794–2.074)	0.309	1.357 (0.815–2.260)	0.454	1.210 (0.720–2.033)	0.472	1.238 (0.747–2.051)	0.407	1.071 (0.641–1.789)	0.795

OR: odds ratio; CI: confidence interval; WC: waist circumference; WHR: waist-to-hip ratio; BMI: body-mass index; TC: total cholesterol; TG: triglyceride; HDL-C: high-density lipoprotein cholesterol; LDL-C: low-density lipoprotein cholesterol; HOMA: homeostatic model assessment; hs-CRP: high-sensitivity C-reactive protein; IgE: immunoglobulin E.

Binary logistic regression analysis allowed us to examine risk factors for either pre-diabetes or diabetes mellitus, compared with variables from the NGG. Before adjustment, hypertension, WC, WHR, hs-CRP, and IgE were significant risk factors for pre-diabetes, while high hypertension, WC, WHR, BMI, HOMA-β index, HOMA-IR index, and hs-CRP levels were significant risk factors for diabetes mellitus (Table S1). After adjusting for age, sex, hypertension, BMI, TC, TG, hyperinsulinemia, hs-CRP, IgE, tryptase, and chymase, only hs-CRP (OR: 3.814 [1.588–9.162, 95% CI], *P* = 0.003) and IgE (OR: 3.367 [1.379–8.197, 95% CI], *P* = 0.008) remained significant risk factors for pre-diabetes, whereas hypertension, WHR, BMI, TC, HOMA-β, HOMA-IR, hs-CRP, and IgE became significant risk factors for diabetes mellitus (Table S1).

### Plasma hs-CRP, IgE, chymase, and tryptase as dependent risk factors of pre-diabetes and diabetes mellitus

As discussed, the inflammatory biomarker hs-CRP associates with diabetes mellitus [7], [15]. When hs-CRP was considered as an interaction variable with all other variables, we found that age, sex, hypertension, WC (or WHR, or BMI), TG, HOMA-β, and IgE were significant risk factors for pre-diabetes and diabetes mellitus, compared with those from the NGG, in an ordinal logistic regression model. In contrast, TC, HDL, LDL, HOMA-IR, tryptase, and chymase were not significant risk factors for pre-diabetes and diabetes mellitus. After adjusting these risk factors using model three (Table 2), both age and sex lost their significance as pre-diabetes and diabetes mellitus risk factors, but other variables remained significant (Table 3). These observations suggest that high plasma hs-CRP, in combination with high plasma IgE, increased the risk of developing pre-diabetes and diabetes mellitus with an odds ratio (OR) of 2.204 (1.020–4.759, 95% CI) before adjustment and an OR of 2.251 (1.037–4.885, 95% CI) after adjustment. We obtained different observations, however, when we used a binary logistic regression model to compare variables from the NGG to those from the PDG or the DMG. Except age and sex, all tested variables (hypertension, WC, WHR, BMI, TC, TG, low HDL, high LDL, hyperinsulinemia, HOMA-β, HOMA-IR, IgE, tryptase, and chymase) were significant risk factors for pre-diabetes, when interacting with high plasma hs-CRP levels before or after adjustment (Table S2). The same sets of variables also were significant risk factors for diabetes mellitus before adjustment. But after adjustment, only hypertension, WHR, TC, high LDL, HOMA-β, HOMA-IR, IgE, and tryptase remained significant risk factors for diabetes mellitus (Table S2).

**Table 3:** Infuence of interactions between CRP and different variables on the risk of developing pre-diabetes and diabetes mellitus — ordinal logistic model.

Variable	Before adjustment		After adjustment (Model three)	
	OR (95.0% CI)	Sig	OR (95.0% CI)	Sig
Age	1.828 (1.027–3.252)	0.040	1.636 (0.909–2.947)	0.101
Sex	1.868 (1.063–3.282)	0.030	1.695 (0.952–3.020)	0.073
Hypertension	2.552 (1.364–4.776)	0.003	2.397 (1.270–4.524)	0.007
WC	2.358 (1.273–4.367)	0.006	2.342 (1.256–4.364)	0.007
WHR	2.690 (1.207–5.998)	0.016	2.631 (1.178–5.876)	0.018
BMI	2.278 (1.202–4.317)	0.012	2.180 (1.135–4.184)	0.019
TC	1.424 (0.583–3.480)	0.438	1.447 (0.589–3.552)	0.420
TG	2.809 (1.335–5.909)	0.006	2.431 (1.118–5.285)	0.025
Lower HDL-C	1.096 (0.302–3.975)	0.889	1.072 (0.295–3.900)	0.916
Higher LDL-C	0.789 (0.185–3.373)	0.750	0.772 (0.180–3.308)	0.727
Hyperinsulinemia	1.829 (0.779–4.296)	0.166	1.550 (0.646–3.718)	0.326
HOMA-β index	4.091 (1.821–9.191)	0.001	4.222 (1.867–9.549)	0.001
HOMA-IR index	2.444 (0.994–6.008)	0.051	2.102 (0.835–5.294)	0.115
IgE	2.204 (1.020–4.759)	0.044	2.251 (1.037–4.885)	0.040
Tryptase	1.853 (0.859–4.000)	0.116	1.698 (0.776–3.713)	0.185
Chymase	2.031 (0.973–4.240)	0.059	1.892 (0.896–3.997)	0.094

OR: odds ratio; CI: confidence interval; WC: waist circumference; WHR: waist-to-hip ratio;

BMI: body-mass index; TC: total cholesterol; TG: triglyceride; HDL-C: high-density lipoprotein cholesterol; LDL-C: low-density lipoprotein cholesterol; HOMA: homeostatic model assessment;

IgE: immunoglobulin E.

Plasma IgE significantly increases in AMI patients, compared with SAP patients or non-CHD patients [19]. In this Chinese population, the interaction of plasma IgE level with hypertension, WC, WHR, BMI, TG, HOMA-β, and chymase showed significant changes in OR, compared with those from the NGG before or after adjustment for all listed diabetes mellitus risk factors (Table 4). This ordinal logistic model of two-variable interactions is important, particularly under complicated circumstances. For example, IgE levels impact the risk of pre-diabetes and diabetes mellitus (OR: 1.674 [1.039–2.698, 95% CI], *P* = 0.034), but chymase alone did not affect the risk of pre-diabetes and diabetes mellitus significantly (OR: 1.387 [0.858–2.244, 95% CI], *P* = 0.182) (Table 2). Interactions of chymase and IgE, however, greatly increased the OR before adjustment (OR: 2.479 [1.079–5.778, 95% CI], *P* = 0.033) and after adjustment (OR: 2.594 [1.118–6.018, 95% CI], *P* = 0.026) for all common diabetes mellitus risk factors (Table 4), suggesting a dependent contribution of plasma chymase and IgE to pre-diabetes and diabetes mellitus.

**Table 4:** Infuence of interactions between IgE and different variables on the risk of developing pre-diabetes and diabetes mellitus — ordinal logistic model.

Variable	Before adjustment		After adjustment (Model three)	
	OR (95.0% CI)	Sig	OR (95.0% CI)	Sig
Age	1.292 (0.709–2.354)	0.402	1.409 (0.762–2.605)	0.275
Sex	1.648 (0.917–2.961)	0.095	1.795 (0.989–3.259)	0.055
Hypertension	2.830 (1.423–5.628)	0.003	2.776 (1.389–5.548)	0.004
WC	2.454 (1.275–4.724)	0.007	2.650 (1.364–5.148)	0.004
WHR	4.015 (1.773–9.093)	0.001	3.994 (1.760–9.063)	0.001
BMI	2.405 (1.246–4.643)	0.009	2.541 (1.303–4.954)	0.006
TC	1.416 (0.555–3.611)	0.467	1.777 (0.675–4.674)	0.244
TG	2.733 (1.362–5.486)	0.005	2.849 (1.403–5.788)	0.004
Lower HDL-C	1.607 (0.337–7.672)	0.552	1.272 (0.395–4.092)	0.687
Higher LDL-C	1.090 (0.161–7.377)	0.930	1.126 (0.165–7.669)	0.903
Hyperinsulinemia	1.642 (0.574–4.695)	0.355	1.633 (0.569–4.689)	0.362
HOMA-β index	4.350 (1.882–10.055)	0.001	5.768 (2.336–14.240)	<0.001
HOMA-IR index	2.672 (0.989–7.213)	0.053	2.738 (1.010–7.428)	0.048
Tryptase	2.091 (0.946–4.625)	0.068	2.167 (0.978–4.802)	0.057
Chymase	2.479 (1.079–5.778)	0.033	2.594 (1.118–6.018)	0.026

OR: odds ratio; CI: confidence interval; WC: waist circumference; WHR: waist-to-hip ratio;

BMI: body-mass index; TC: total cholesterol; TG: triglyceride; HDL-C: high-density lipoprotein cholesterol; LDL-C: low-density lipoprotein cholesterol; HOMA: homeostatic model assessment.

When we performed binary logistic regression analysis, we found that interactions of high IgE with hypertension, WC, WHR, TC, low HDL, high LDL, hyperinsulinemia, HOMA-β, HOMA-IR, and chymase significantly increased the risk of having pre-diabetes before and after adjustment, compared with those from the NGG (Table S3). When we compared the variables between the DMG and the NGG, we found that interactions between IgE and hypertension, WC, WHR, BMI, TC, TG, HOMA-β, and HOMA-IR were significant risk factors for diabetes mellitus. After adjustment, while interactions between IgE and hypertension, WHR, BMI, TC, HOMA-β, and HOMA-IR remained significant risk factors for diabetes mellitus, the interactions of IgE with low HDL, high LDL, chymase, and tryptase significantly increased the risk of having diabetes mellitus (Table S3).

We made similar conclusions after analyzing interactions between chymase and several other diabetes mellitus factors, including hypertension, WC, WHR, TG, HOMA-β and HOMA-IR (Table 5). Under all methods tested, however, plasma tryptase alone did not correlate with plasma glucose levels (Table 1), nor did it affect the risk of pre-diabetes and diabetes mellitus (OR: 1.283 [0.794–2.074, 95% CI], *P* = 0.309) (Table 2). Its interactions with hs-CRP (OR: 1.853 [0.859–4.000, 95% CI], *P* = 0.116) (Table 3), IgE (2.091 [0.946–4.625, 95% CI], *P* = 0.068) (Table 4), or chymase (OR: 1.220 [0.566–2.629], 95% CI], *P* = 0.612) (Table 5) did not significantly influence the risk of developing pre-diabetes and diabetes mellitus. Interactions of plasma tryptase with hypertension, WC, WHR, TC, TG, HOMA-β and HOMA-IR did, however, increase the OR in an ordinal logistic model significantly (Table 6).

**Table 5:** Infuence of interactions between chymase and different variables on the risk of developing pre-diabetes and diabetes mellitus — ordinal logistic model.

Variable	Before adjustment		After adjustment (Model three)	
	OR (95.0% CI)	Sig	OR (95.0% CI)	Sig
Age	1.328 (0.721–2.543)	0.351	1.291 (0.707–2.355)	0.406
Sex	1.614 (0.903–2.884)	0.106	1.542 (0.858–2.771)	0.148
Hypertension	2.388 (1.235–4.425)	0.009	2.281 (1.205–4.319)	0.011
WC	2.355 (1.168–4.479)	0.017	2.453 (1.213–4.962)	0.013
WHR	3.254 (1.351–7.838)	0.009	3.311 (1.374–7.983)	0.008
BMI	1.707 (0.864–3.371)	0.124	1.760 (0.889–3.483)	0.104
TC	2.206 (0.972–5.005)	0.058	2.275 (1.001–5.173)	0.050
TG	2.033 (1.006–4.110)	0.048	1.861 (0.913–3.795)	0.087
Lower HDL-C	1.490 (0.349–6.361)	0.590	1.507 (0.353–6.433)	0.579
Higher LDL-C	1.510 (0.316–7.209)	0.605	1.527 (0.320 –7.289)	0.595
Hyperinsulinemia	1.988 (0.763–5.184)	0.160	1.955 (0.749–5.100)	0.171
HOMA-β index	6.682 (2.267–16.996)	<0.001	7.078 (2.772–18.074)	<0.001
HOMA-IR index	4.033 (1.485–10.955)	0.006	3.969 (1.460–10.785)	0.007
Tryptase	1.220 (0.566–2.629)	0.612	1.223 (0.567–2.638)	0.608

OR: odds ratio; CI: confidence interval; WC: waist circumference; WHR: waist-to-hip ratio;

BMI: body-mass index; TC: total cholesterol; TG: triglyceride; HDL-C: high-density lipoprotein cholesterol; LDL-C: low-density lipoprotein cholesterol; HOMA: homeostatic model assessment.

**Table 6:** Infuence of interactions between tryptase and different variables on the risk of developing pre-diabetes and diabetes mellitus — ordinal logistic model.

Variables	Before adjustment		After adjustment (Model three)	
	OR (95.0% CI)	Sig	OR (95.0% CI)	Sig
Age	1.241 (0.691–2.228)	0.470	1.210 (0.668–2.193)	0.530
Sex	1.014 (0.557–1.846)	0.963	0.953 (0.518–1.753)	0.876
Hypertension	2.656 (1.357–5.198)	0.004	2.585 (1.320–5.061)	0.006
WC	2.157 (1.084–4.295)	0.029	2.165 (1.086–4.314)	0.028
WHR	3.642 (1.563–8.489)	0.003	3.696 (1.584–8.623)	0.002
BMI	1.560 (0.800–3.042)	0.192	1.508 (0.768–2.961)	0.232
TC	2.692 (1.104–6.564)	0.029	2.773 (1.135–6.775)	0.025
TG	2.351 (1.172–4.714)	0.016	2.158 (1.068–4.360)	0.032
Lower HDL-C	1.259 (0.391–4.049)	0.700	1.272 (0.395–4.092)	0.687
Higher LDL-C	1.312 (0.808–2.131)	0.272	1.289 (0.789–2.106)	0.310
Hyperinsulinemia	1.383 (0.543–3.523)	0.497	1.184 (0.453–3.095)	0.731
HOMA-β index	5.035 (2.094–12.107)	<0.001	5.768 (2.336–14.240)	<0.001
HOMA-IR index	2.747 (1.098–6.871)	0.031	2.406 (0.940–6.156)	0.067

OR: odds ratio; CI: confidence interval; WC: waist circumference; WHR: waist-to-hip ratio;

BMI: body-mass index; TC: total cholesterol; TG: triglyceride; HDL-C: high-density lipoprotein cholesterol; LDL-C: low-density lipoprotein cholesterol; HOMA: homeostatic model assessment.

When the NGG was compared independently with the PDG in a binary logistic model, we found that interactions between chymase and hypertension, WC, WHR, BMI, LDL, hyperinsulinemia, and tryptase were significant risk factors for pre-diabetes. After adjustment, interactions between chymase and WC, WHR, or HOMA-IR index affected the risk of pre-diabetes significantly (Table S4). When the NGG was compared with the DMG in the same model, interactions between chymase and hypertension, WHR, BMI, HOMA-β, HOMA-IR, and tryptase were significant risk factors for diabetes mellitus before and after adjustment (Table S4). When interactions between tryptase and other variables were considered in the binary logistic regression analysis, WC was the only significant risk factor for pre-diabetes before adjustment, and it lost its significance after adjustment (Table S5). When the NGG was compared with the DMG, however, interactions between tryptase and obesity variables (WC, WHR, and BMI), HOMA-β, and HOMA-IR were significant risk factors for diabetes mellitus, and BMI, HOMA-β, and HOMA-IR remained significant risk factors for diabetes mellitus after adjustment (Table S5).

## Discussion

Accumulating evidence indicates that age, sex, obesity, hypertension, dyslipidemia, insulin resistance, decreased β-cell sensitivity, hyperinsulinemia, HbA1c, and hs-CRP are related to pre-diabetes and diabetes mellitus [25]–[36]. Inflammatory cytokines are important to the pathogenesis of pre-diabetes and type 2 DM [37]–[39]. This study reveals that both IgE and chymase correlate with glucose tolerance in humans. Interactions with other diabetes risk factors greatly increased the impact of these mast-cell–related molecules on pre-diabetes and diabetes mellitus.

Mast cell chymase and tryptase are important proteases that have been implicated in cardiovascular diseases. Patients with atherosclerosis and patients with abdominal aortic aneurysms (AAAs) have elevated levels of these proteases [11], [12], [18]. Mechnistically, chymase contributes to angiogenesis, SMC apoptosis, and mast cell cysteinyl cathepsin expression. Mast cells lacking chymase or SMCs that are treated with chymase-deficient mast cells demonstrate reduced cysteinyl cathepsin expression and activties [11]. In contrast, mast cell tryptase contributes to EC chemokine expression, monocyte and mast cell cysteinyl cathepsin expression, leukocyte migration, and SMC apoptosis [12]. In patients with small AAAs, plasma levels of these proteases associate with abdominal aorta expansion rate, risks of later surgical repair, or overall mortality. Although a direct participation of chymase or tryptase in obesity or diabetes has not been tested in any experimental model, cysteinyl protease cathepsins — which are indirectly regulated by chymase and tryptase [11], [12] — have been confirmed in diet-induced obesity and diabetes and in genetically-generated obesity and diabetes. Mice deficient in cathepsin L or cathepsin K, or treated with their selective inhibitors, are leaner than control mice or have significantly improved glucose sensitivity [40], [41]. Both chymase and tryptase, therefore, may directly or indirectly participate in the pathogenesis of diabetes mellitus — a hypothesis that merits further investigation.

IgE often associates with allergic responses, but several small human population studies indicated an association between serum IgE levels and CHD. In the Helsinki Heart Study [42], a nested case-control design and logistic regression analysis of 135 patients with CHD and 135 control subjects, serum IgE levels were higher in CHD patients than in control subjects. In a group of 13 stable patients from Turkey who underwent elective coronary artery stenting, serum levels of IgE and hs-CRP reached their peaks at the second and third post-procedure days, but returned to the baseline at the end of the first month [43]. IgE, like hs-CRP, might serve as a biomarker of inflammation in CHD, as shown in a recent study from a group in Pakistan: 99 patients with a history of ischemic heart disease (IHD) and established atherosclerosis on angiography showed increased serum levels of oxidized LDL and IgE, compared with those in 101 age-matched and sex-matched healthy subjects without a known history of IHD [44]. We demonstrated in two independent Chinese populations (982 and 240 patients, respectively) that plasma IgE levels were highest among AMI patients, followed by UAP patients, SAP patients, and non-CHD volunteers [19]. Although our original study [19] tested the role of IgE on macrophages, SMCs, and ECs, one conventional function of IgE is the activation of mast cells — a cell type that plays critical roles in type 2 DM [10]. Our current study demonstrates that plasma IgE levels strongly correlate with glucose tolerance status (Table 1) and serves as a strong risk factor for pre-diabetes and diabetes mellitus, either independently or in their interactions with mast cell protease or many other diabetes risk factors (Table 2, Table S1). Combining these clinical data and our recent discoveries of IgE function in atherosclerosis [19], we conjecture that IgE participates directly in the pathogenesis of diabetes mellitus — a hypothesis that can be tested in animals in the future.

But this study also has limitations. When we enrolled these patients and volunteers, we did not exclude those who might have asthma or other allergic diseases, which might affect blood IgE or mast cell protease levels. Ideally, their circulating eosinophilic leukocyte contents would be measured. A larger and more complete population study therefore may be required to affirm our observations. Nevertheless, this study provided the first evidence that elevated plasma levels of mast cell proteases and IgE may serve as important risk factors for human type 2 DM, particularly when hs-CRP or other common diabetes mellitus risk factors are considered. These mast cell proteases or IgE also may serve as biomarkers for human type 2 DM.

## Supporting Information

Table S1 **Influence of different variables on the relative risk of developing pre-diabetes and diabetes mellitus.**
(DOC)

Table S2 **Infuence of interactions between CRP and different variables on the relative risk of developing pre-diabetes and diabetes mellitus.**
(DOC)

Table S3 **Infuence of interactions between IgE and different variables on the relative risk of developing pre-diabetes and diabetes mellitus.**
(DOC)

Table S4 **Infuence of interactions between chymase and different variables on the relative risk of developing pre-diabetes and diabetes mellitus.**
(DOC)

Table S5 **Infuence of interactions between tryptase and different variables on the relative risk of developing pre-diabetes and diabetes mellitus.**
(DOC)
