# Supplementary material for: Immunoglobulin E and Mast Cell Proteases Are Potential Risk Factors of Human Pre-Diabetes and Diabetes Mellitus
Source: PLoS One. 2011 Dec 16;6(12):e28962. doi: 10.1371/journal.pone.0028962 (PMC3241693; doi:10.1371/journal.pone.0028962)
Supplement: Table S1 — Influence of different variables on the relative risk of developing pre-diabetes and diabetes mellitus. (DOC) [file pone.0028962.s001.doc]

**Table S1**

**Immunoglobulin E and mast cell proteases are potential risk factors of human pre-diabetes and diabetes mellitus**

Zhen Wang, Hong Zhang, Xu-Hui Shen, Kui-Li Jin, Guo-fen Ye, Li Qian, Bo Li, Yong-Hong Zhang, Guo-Ping Shi

**Table S1.** Influence of different variables on the relative risk of developing pre-diabetes and diabetes mellitus.*

| **Variable** | **NGG versus PDG** | | | | **NGG versus DMG** | | | |
| --- | --- | --- | --- | --- | --- | --- | --- | --- |
|  | **Before adjustment** | | **After adjustment**** | | **Before adjustment** | | **After adjustment**** | |
|  | **OR (95.0% CI)** | **Sig*** | **OR (95.0% CI)** | **Sig*** | **OR (95.0% CI)** | **Sig*** | **OR (95.0% CI)** | **Sig*** |
| Age | 0.975 (0.556-1.710) | 0.093 | 0.734 (0.386-1.399) | 0.347 | 0.893 (0.469-1.701) | 0.731 | 0.501 (0.224-1.121) | 0.092 |
| Sex | 0.576 (0.302-1.100) | 0.059 | 0.540 (0.259-1.124) | 0.099 | 1.312 (0.625-2.755) | 0.473 | 0.605 (0.246-1.488) | 0.274 |
| Hypertension | 1.776 (1.015-3.106) | 0.044 | 1.429 (0.763-2.674) | 0.265 | 1.740 (1.427-5.263) | 0.002 | 2.506 (1.170-5.376) | 0.018 |
| WC | 2.183 (1.250-3.802) | 0.006 | 2.347 (0.950-5.780) | 0.065 | 2.132 (1.111-4.098) | 0.023 | 1.319 (0.499-3.497) | 0.576 |
| WHR | 3.457 (1.404-8.513) | 0.007 | 2.208 (0.816-5.952) | 0.119 | 5.952 (2.283-15.625) | <0.001 | 4.329 (1.418-13.158) | 0.010 |
| BMI | 1.618 (0.930-2.817) | 0.089 | 1.149 (0.595-2.222) | 0.678 | 2.020 (1.066-3.831) | 0.031 | 2.604 (1.181-5.747) | 0.018 |
| TC | 1.493 (0.736-3.021) | 0.267 | 1.536 (0.676-3.484) | 0.306 | 1.695 (0.780-3.690) | 0.182 | 2.597 (1.026-6.579) | 0.044 |
| TG | 1.403 (0.783-2.506) | 0.256 | 1.188 (0.610-2.309) | 0.613 | 1.802 (0.943-3.448) | 0.074 | 0.993 (0.450-2.188) | 0.986 |
| Lower HDL-C | 1.002 (0.376-2.674) | 0.997 | 0.917 (0.297-2.825) | 0.879 | 0.933 (0.311-2.793) | 0.901 | 0.883 (0.238-3.279) | 0.853 |
| Higher LDL-C | 0.211 (0.027-1.650) | 0.138 | 2.247 (0.225-22.222) | 0.491 | 2.882 (0.294-28.571) | 0.364 | 1.739 (0.129-23.256) | 0.677 |
| Hyperinsulinemia | 1.504 (0.772-2.933) | 0.230 | 1.497 (0.683-3.279) | 0.313 | 1.312 (0.625-2.755) | 0.473 | 0.794 (0.314-2.008) | 0.627 |
| HOMA-β index | 0.801 (0.382-1.681) | 0.558 | 1.605 (0.652-3.953) | 0.303 | 5.076 (2.381-10.753) | <0.001 | 16.667 (5.348-52.632) | <0.001 |
| HOMA-IR index | 2.033 (0.964-4.292) | 0.062 | 3.802 (0.910-15.873) | 0.067 | 3.030 (1.408-6.536) | 0.005 | 3.074 (2.087-5.267) | <0.001 |
| Hs-CRP | 4.390 (1.900-10.151) | 0.001 | 3.814 (1.588-9.162) | 0.003 | 2.952 (1.266-6.886) | 0.012 | 2.647 (1.016-6.892) | 0.046 |
| IgE | 2.688 (1.245-5.780) | 0.012 | 3.367 (1.379-8.197) | 0.008 | 1.923 (0.891-4.149) | 0.095 | 2.976 (1.157-7.692) | 0.024 |
| Chymase | 1.059 (0.944-4.000) | 0.071 | 1.757 (0.798-3.861) | 0.162 | 2.008 (0.890-4.525) | 0.093 | 1.745 (0.689-4.425) | 0.241 |
| Tryptase | 1.120 (0.576-2.174) | 0.739 | 0.799 (0.380-1.678) | 0.553 | 1.437 (0.689-3.003) | 0.334 | 1.299 (0.545-3.096) | 0.555 |

NGG: normal glucose group; PDG: pre-diabetes group; DMG: diabetes mellitus group; OR: odds ratio; CI: confidence interval; WC: waist circumference; WHR: waist-to-hip ratio; BMI: body-mass index; TC: total cholesterol; TG: triglyceride; HDL-C: high-density lipoprotein cholesterol; LDL-C: low-density lipoprotein cholesterol; HOMA: homeostatic model assessment; IgE: immunoglobulin E.

*Binary logistic model. **Adjusted for age, sex, hypertension, BMI, TC, TG, hyperinsulinemia, hs-CRP, IgE, tryptase, and chymase.
