# Supplementary material for: Immunoglobulin E and Mast Cell Proteases Are Potential Risk Factors of Human Pre-Diabetes and Diabetes Mellitus
Source: PLoS One. 2011 Dec 16;6(12):e28962. doi: 10.1371/journal.pone.0028962 (PMC3241693; doi:10.1371/journal.pone.0028962)
Supplement: Table S2 — Infuence of interactions between CRP and different variables on the relative risk of developing pre-diabetes and diabetes mellitus. (DOC) [file pone.0028962.s002.doc]

**Table S2**

**Immunoglobulin E and mast cell proteases are potential risk factors of human pre-diabetes and diabetes mellitus**

Zhen Wang, Hong Zhang, Xu-Hui Shen, Kui-Li Jin, Guo-fen Ye, Li Qian, Bo Li, Yong-Hong Zhang, Guo-Ping Shi

**Table S2.** Infuence of interactions between CRP and different variables on the relative risk of developing pre-diabetes and diabetes mellitus.*

| **Variable** | **NGG versus PDG** | | | | **NGG versus DMG** | | | |
| --- | --- | --- | --- | --- | --- | --- | --- | --- |
|  | **Before adjustment** | | **After adjustment****  **(Model three)** | | **Before adjustment** | | **After adjustment****  **(Model three)** | |
|  | **OR (95.0% CI)** | **Sig*** | **OR (95.0% CI)** | **Sig*** | **OR (95.0% CI)** | **Sig*** | **OR (95.0% CI)** | **Sig*** |
| Age | 1.421 (0.794-0.545) | 0.237 | 0.580 (0.284-1.184) | 0.135 | 1.165 (0.597-2.273) | 0.655 | 0.724 (0.326-1.607) | 0.427 |
| Sex | 0.856 (0.426-1.720) | 0.663 | 0.764 (0.331-1.760) | 0.527 | 0.908 (0.400-2.059) | 0.817 | 0.674 (0.254-1.789) | 0.429 |
| Hypertension | 2.523 (1.442 -4.414) | 0.001 | 2.238 (1.217-4.115) | 0.010 | 3.353 (1.708-6.581) | <0.001 | 3.230 (1.488-7.013) | 0.003 |
| WC | 2.642 (1.499-4.656) | 0.001 | 2.547 (1.153-5.625) | 0.021 | 2.217 (1.138-4.319) | 0. 019 | 1.389 (0.580-3.326) | 0.461 |
| WHR | 4.460 (2.250-8.844) | <0.001 | 3.130 (1.434-6.833) | 0.001 | 4.788 (2.302-9.957) | <0.001 | 4.144 (1.790-9.594) | 0.001 |
| BMI | 2.136 (1.218-3.744) | 0.008 | 1.546 (0.826-2.892) | 0.173 | 2.142 (1.113-4.122) | 0.023 | 1.967 (1.894-4.329) | 0.093 |
| TC | 2.917 (1.576-5.399) | 0.001 | 2.983 (1.521-5.851) | 0.001 | 2.870 (1.467-5.615) | 0.002 | 3.697 (1.643-8.318) | 0.002 |
| TG | 2.567 (1.464-4.502) | 0.001 | 2.007 (1.083-3.721) | 0.027 | 2.223 (1.174-4.209) | 0.014 | 1.745 (0.828-3.676) | 0.143 |
| Lower HDL-C | 2.917 (1.467-5.801) | 0.002 | 2.668 (1.285-5.542) | 0.008 | 2.470 (1.182-5.159) | 0.016 | 1.985 (0.825-4.774) | 0.126 |
| Higher LDL-C | 5.063 (2.196-11.673) | <0.001 | 4.293 (1.793-10.277) | 0.001 | 3.519 (1.523-8.133) | 0.003 | 3.347 (1.303-8.599) | 0.012 |
| Hyperinsulinemia | 2.605 (1.443-4.703) | 0.001 | 2.416 (1.253-4.659) | 0.008 | 2.036 (1.057-3.923) | 0.034 | 1.481 (0.690-3.178) | 0.313 |
| HOMA-β index | 2.375 (1.281-4.403) | 0.006 | 2.688 (1.344-5.375) | 0.005 | 4.924 (2.484-9.763) | <0.001 | 9.316 (3.626-23.935) | <0.001 |
| HOMA-IR index | 3.329 (1.780-6.227) | <0.001 | 4.083 (1.781-9.363) | 0.001 | 4.059 (2.051-8.033) | <0.001 | 5.943 (2.176-16.225) | 0.001 |
| IgE | 3.733 (1.973-7.063) | <0.001 | 4.283 (2.127-8.626) | <0.001 | 2.786 (1.414-5.487) | 0.003 | 4.159 (1.830-9.451) | 0.001 |
| Tryptase | 2.366 (1.295-4.324) | 0.005 | 1.926 (1.014-3.658) | 0.045 | 2.240 (1.146-4.380) | 0.018 | 2.492 (1.152-5.393) | 0.020 |
| Chymase | 2.812 (1.513-5.227) | 0.001 | 2.697 (1.387-5.242) | 0.003 | 2.325 (1.174-4.607) | 0.016 | 2.450 (0.850-7.061) | 0.097 |

NGG: normal glucose group; PDG: pre-diabetes group; DMG: diabetes mellitus group; OR: odds ratio; CI: confidence interval; WC: waist circumference; WHR: waist-to-hip ratio; BMI:

body-mass index; TC: total cholesterol; TG: triglyceride; HDL-C: high-density lipoprotein cholesterol; LDL-C: low-density lipoprotein cholesterol; HOMA: homeostatic model assessment;

IgE: immunoglobulin E.

*Binary logistic model. **Adjusted for age, sex, hypertension, BMI, TC, TG, hyperinsulinemia, hs-CRP, IgE, tryptase, and chymase.
