# Supplementary material for: Immunoglobulin E and Mast Cell Proteases Are Potential Risk Factors of Human Pre-Diabetes and Diabetes Mellitus
Source: PLoS One. 2011 Dec 16;6(12):e28962. doi: 10.1371/journal.pone.0028962 (PMC3241693; doi:10.1371/journal.pone.0028962)
Supplement: Table S3 — Infuence of interactions between IgE and different variables on the relative risk of developing pre-diabetes and diabetes mellitus. (DOC) [file pone.0028962.s003.doc]

**Table S3**

**Immunoglobulin E and mast cell proteases are potential risk factors of human pre-diabetes and diabetes mellitus**

Zhen Wang, Hong Zhang, Xu-Hui Shen, Kui-Li Jin, Guo-fen Ye, Li Qian, Bo Li, Yong-Hong Zhang, Guo-Ping Shi

**Table S3.** Infuence of interactions between IgE and different variables on the relative risk of developing pre-diabetes and diabetes mellitus.*

| **Variable** | **NGG versus PDG** | | | | **NGG versus DMG** | | | |
| --- | --- | --- | --- | --- | --- | --- | --- | --- |
|  | **Before adjustment** | | **After adjustment ****  **(Model three)** | | **Before adjustment** | | **After adjustment ****  **(Model three)** | |
|  | **OR (95.0% CI)** | **Sig*** | **OR (95.0% CI)** | **Sig*** | **OR (95.0% CI)** | **Sig*** | **OR (95.0% CI)** | **Sig*** |
| Age | 1.324 (0.742-2.363) | 0.342 | 1.181 (0.622-2.242) | 0.612 | 1.486 (0.748-2.950) | 0.258 | 1.114 (0.505-2.457) | 0.789 |
| Sex | 0.862 (0.429-1.731) | 0.676 | 0.876 (0.412-1.859) | 0.730 | 1.080 (0.465-2.506) | 0.859 | 0.899 (0.344-2.353) | 0.829 |
| Hypertension | 2.269 (1.301-3.957) | 0.004 | 2.278 (1.224-4.237) | 0.009 | 4.082 (2.008-8.333) | <0.001 | 4.673 (2.075-10.526) | <0.001 |
| WC | 2.586 (1.466-4.562) | 0.001 | 3.165 (1.374-7.299) | 0.007 | 2.273 (1.155-4.464) | 0.018 | 2.146 (0.897-5.128) | 0.086 |
| WHR | 2.944 (1.553-5.578) | 0.001 | 2.825 (1.370-5.814) | 0.005 | 2.924 (1.473-5.814) | 0.002 | 3.300 (1.471-7.407) | 0.004 |
| BMI | 1.952 (1.117-3.412) | 0.019 | 1.504 (0.803-2.817) | 0.202 | 2.288 (1.179-4.444) | 0.014 | 3.356 (1.499-7.519) | 0.003 |
| TC | 2.189 (1.203-3.984) | 0.010 | 2.457 (1.255-4.808) | 0.009 | 2.364 (1.227-4.545) | 0.010 | 3.497 (1.597-7.634) | 0.002 |
| TG | 1.480 (0.855-2.564) | 0.161 | 1.462 (0.792-2.695) | 0.225 | 1.795 (0.951-3.378) | 0.071 | 1.595 (0.777-3.268) | 0.203 |
| Lower HDL-C | 1.911 (1.003-3.642) | 0.049 | 2.188 (1.046-4.587) | 0.038 | 1.672 (0.840-3.333) | 0.144 | 2.364 (1.016-5.495) | 0.046 |
| Higher LDL-C | 2.907 (1.394-6.062) | 0.004 | 3.236 (1.391-7.519) | 0.006 | 2.092 (0.995-4.405) | 0.052 | 2.841 (1.155-6.993) | 0.023 |
| Hyperinsulinemia | 3.733 (1.973-7.063) | <0.001 | 2.066 (1.072-3.984) | 0.030 | 1.845 (0.976-3.497) | 0.060 | 1.695 (0.811-3.546) | 0.160 |
| HOMA-β index | 2.252 (1.215-4.177) | 0.010 | 2.801 (1.372-5.714) | 0.005 | 4.608 (2.336-9.091) | <0.001 | 14.085 (4.902-40.000) | <0.001 |
| HOMA-IR index | 2.408 (1.324-4.382) | 0.004 | 3.257 (1.453-7.353) | 0.004 | 3.247 (1.681-6.289) | <0.001 | 6.061 (2.364-15.625) | <0.001 |
| Chymase | 2.292 (1.255-4.188) | 0.007 | 2.288 (1.174-4.464) | 0.015 | 1.869 (0.966-3.623) | 0.063 | 2.174 (1.021-4.630) | 0.044 |
| Tryptase | 1.748 (0.969-3.153) | 0.064 | 1.560 (0.818-1.560) | 0.177 | 1.736 (0.904-3.333) | 0.097 | 2.128 (0.987-4.587) | 0.054 |

NGG: normal glucose group; PDG: pre-diabetes group; DMG: diabetes mellitus group; OR: odds ratio; CI: confidence interval; WC: waist circumference; WHR: waist-to-hip ratio;

BMI: body-mass index; TC: total cholesterol; TG: triglyceride; HDL-C: high-density lipoprotein cholesterol; LDL-C: low-density lipoprotein cholesterol; HOMA: homeostatic model

assessment; IgE: immunoglobulin E.

*Binary logistic model. **Adjusted for age, sex, hypertension, BMI, TC, TG, hyperinsulinemia, hs-CRP, IgE, tryptase, and chymase.
