# Supplementary material for: Immunoglobulin E and Mast Cell Proteases Are Potential Risk Factors of Human Pre-Diabetes and Diabetes Mellitus
Source: PLoS One. 2011 Dec 16;6(12):e28962. doi: 10.1371/journal.pone.0028962 (PMC3241693; doi:10.1371/journal.pone.0028962)
Supplement: Table S4 — Infuence of interactions between chymase and different variables on the relative risk of developing pre-diabetes and diabetes mellitus. (DOC) [file pone.0028962.s004.doc]

**Table S4**

**Immunoglobulin E and mast cell proteases are potential risk factors of human pre-diabetes and diabetes mellitus**

Zhen Wang, Hong Zhang, Xu-Hui Shen, Kui-Li Jin, Guo-fen Ye, Li Qian, Bo Li, Yong-Hong Zhang, Guo-Ping Shi

**Table S4.** Infuence of interactions between chymase and different variables on the relative risk of developing pre-diabetes and diabetes mellitus.*

| **Variable** | **NGG versus PDG** | | | | **NGG versus DMG** | | | |
| --- | --- | --- | --- | --- | --- | --- | --- | --- |
|  | **Before adjustment** | | **After adjustment****  **(Model three)** | | **Before adjustment** | | **After adjustment****  **(Model three)** | |
|  | **OR (95.0% CI)** | **Sig*** | **OR (95.0% CI)** | **Sig*** | **OR (95.0% CI)** | **Sig*** | **OR (95.0% CI)** | **Sig*** |
| Age | 1.309 (0.727-2.358) | 0.370 | 0.921 (0.478-1.773) | 0.805 | 1.145 (0.583-2.252) | 0.693 | 0.601 (0.267-1.350) | 0.217 |
| Sex | 0.709 (0.340-1.477) | 0.359 | 0.629 (0.283-1.399) | 0.255 | 0.787 (0.344-1.799) | 0.570 | 0.580 (0.220-1.524) | 0.269 |
| Hypertension | 1.852 (1.055-3.247) | 0.032 | 1.565 (0.839-2.924) | 0.159 | 2.415 (1.235-4.717) | 0.010 | 2.439 (1.129-5.263) | 0.023 |
| WC | 2.933 (1.637-5.263) | <0.001 | 2.747 (1.229-6.135) | 0.014 | 2.591 (1.311-5.128) | 0.006 | 1.866 (0.759-4.587) | 0.174 |
| WHR | 2.611 (1.389-4.902) | 0.003 | 2.053 (1.022-4.115) | 0.043 | 3.472 (1.704-7.092) | 0.001 | 3.226 (1.389-7.519) | 0.006 |
| BMI | 2.237 (1.264-3.968) | 0.006 | 1.686 (0.890-3.205) | 0.109 | 2.564 (1.316-4.975) | 0.006 | 3.257 (1.473-7.194) | 0.004 |
| TC | 1.497 (0.833-2.688) | 0.177 | 1.376 (0.716-2.646) | 0.339 | 1.692 (0.872-3.279) | 0.120 | 1.757 (0.807-3.831) | 0.155 |
| TG | 1.715 (0.978-3.012) | 0.060 | 1.453 (0.779-2.710) | 0.240 | 2.033 (1.067-3.876) | 0.031 | 1.120 (0.515-2.433) | 0.776 |
| Lower HDL-C | 1.650 (0.873-3.125) | 0.123 | 1.511 (0.748-3.049) | 0.250 | 1.587 (0.775-3.247) | 0.207 | 1.458 (0.624-3.401) | 0.384 |
| Higher LDL-C | 2.096 (1.046-4.202) | 0.037 | 0.998 (0.089-11.111) | 0.998 | 2.049 (0.935-4.505) | 0.073 | 1.634 (0.664-4.032) | 0.285 |
| Hyperinsulinemia | 1.894 (1.063-3.378) | 0.030 | 1.773 (0.923-3.401) | 0.086 | 1.493 (0.776-2.874) | 0.230 | 0.946 (0.428-2.088) | 0.890 |
| HOMA-β index | 1.477 (0.822-2.653) | 0.192 | 1.618 (0.824-3.175) | 0.162 | 3.546 (1.815-6.944) | <0.001 | 12.346 (4.016-37.037) | <0.001 |
| HOMA-IR index | 0.487 (0.215-1.101) | 0.084 | 2.688 (1.221-5.917) | 0.014 | 2.809 (1.435-5.495) | 0.003 | 2.959 (1.136-7.692) | 0.026 |
| Tryptase | 1.835 (1.010-3.333) | 0.046 | 1.445 (0.754-2.770) | 0.267 | 2.132 (1.089-4.167) | 0.027 | 2.222 (1.021-4.831) | 0.044 |

NGG: normal glucose group; PDG: pre-diabetes group; DMG: diabetes mellitus group; OR: odds ratio; CI: confidence interval; WC: waist circumference; WHR: waist-to-hip ratio; BMI:

body-mass index; TC: total cholesterol; TG: triglyceride; HDL-C: high-density lipoprotein cholesterol; LDL-C: low-density lipoprotein cholesterol; HOMA: homeostatic model assessment;

IgE: immunoglobulin E.

*Binary logistic model. **Adjusted for age, sex, hypertension, BMI, TC, TG, hyperinsulinemia, hs-CRP, IgE, tryptase, and chymase.
