# Supplementary material for: Immunoglobulin E and Mast Cell Proteases Are Potential Risk Factors of Human Pre-Diabetes and Diabetes Mellitus
Source: PLoS One. 2011 Dec 16;6(12):e28962. doi: 10.1371/journal.pone.0028962 (PMC3241693; doi:10.1371/journal.pone.0028962)
Supplement: Table S5 — Infuence of interactions between tryptase and different variables on the relative risk of developing pre-diabetes and diabetes mellitus. (DOC) [file pone.0028962.s005.doc]

**Table S5**

**Immunoglobulin E and mast cell proteases are potential risk factors of human pre-diabetes and diabetes mellitus**

Zhen Wang, Hong Zhang, Xu-Hui Shen, Kui-Li Jin, Guo-fen Ye, Li Qian, Bo Li, Yong-Hong Zhang, Guo-Ping Shi

**Table S5.** Infuence of interactions between tryptase and different variables on the relative risk of developing pre-diabetes and diabetes mellitus.*

| **Variable** | **NGG versus PDG** | | | | **NGG versus DMG** | | | |
| --- | --- | --- | --- | --- | --- | --- | --- | --- |
|  | **Before adjustment** | | **After adjustment****  **(Model three)** | | **Before adjustment** | | **After adjustment****  **(Model three)** | |
|  | **OR (95.0% CI)** | **Sig*** | **OR (95.0% CI)** | **Sig*** | **OR (95.0% CI)** | **Sig*** | **OR (95.0% CI)** | **Sig*** |
| Age | 1.244 (0.692 -2.237) | 0.466 | 0.861 (0.448-1.653) | 0.651 | 1.082 (0.552 -2.119) | 0.819 | 0.666 (0.301-1.473) | 0.316 |
| Sex | 0.840 (0.410 -1.724) | 0.635 | 0.751(0.342 -1.647) | 0.475 | 1.115 (0.466-2.667) | 0.807 | 0.789 (0.283 -2.198) | 0.650 |
| Hypertension | 1.346 (0.766-2.370) | 0.301 | 1.045 (0.556 -1.965) | 0.890 | 1.949 (0.992 -3.846) | 0.053 | 1.931 (0.876 -4.255) | 0.103 |
| WC | 2.257 (1.264 -4.032) | 0.006 | 1.832 (0.848 -3.953) | 0.123 | 2.525 (1.259 -5.051) | 0.009 | 1.664 (0.693-3.984) | 0.255 |
| WHR | 1.650 (0.907 -3.003) | 0.101 | 1.089 (0.552 -2.151) | 0.805 | 2.415 (1.227 -4.739) | 0.011 | 2.033 (0.923-4.484) | 0.079 |
| BMI | 1.538 (0.873 -2.717) | 0.136 | 1.082 (0.566-2.070) | 0.812 | 2.469 (1.252 -4.878) | 0.009 | 2.976 (1.344-6.623) | 0.007 |
| TC | 1.232 (0.694-2.183) | 0.477 | 0.937 (0.496 -1.770) | 0.841 | 1.395 (0.729 -2.667) | 0.315 | 1.441 (0.676 -3.067) | 0.345 |
| TG | 1.239 (0.710 -2.160) | 0.450 | 0.915 (0.492 -1.701) | 0.778 | 1.546 (0.816 -2.933) | 0.182 | 0.945 (0.442 -2.020) | 0.884 |
| Lower HDL-C | 1.074 (0.580 -1.988) | 0.819 | 0.833 (0.421 -1.650) | 0.601 | 1.350 (0.676 -2.695) | 0.396 | 1.269 (0.557 -2.890) | 0.571 |
| Higher LDL-C | 1.425 (0.751 -2.703) | 0.279 | 0.900 (0.433 -1.869) | 0.777 | 1.587 (0.775 -3.247) | 0.207 | 1.351 (0.581-3.145) | 0.484 |
| Hyperinsulinemia | 1.346 (0.763 -2.375) | 0.305 | 1.101(0.577 -2.101) | 0.771 | 1.453 (0.763 -2.770) | 0.255 | 1.196 (0.557 -2.571) | 0.646 |
| HOMA-β index | 1.038 (0.584-1.845) | 0.899 | 0.941 (0.484 -1.832) | 0.858 | 3.106 (1.600 -6.024) | 0.001 | 5.525 (2.247-13.514) | <0.001 |
| HOMA-IR index | 1.684 (0.939 -3.021) | 0.080 | 1.335 (0.613-2.915) | 0.467 | 2.564 (1.326 -4.975) | 0.005 | 3.021 (1.235-7.353) | 0.015 |

NGG: normal glucose group; PDG: pre-diabetes group; DMG: diabetes mellitus group; OR: odds ratio; CI: confidence interval; WC: waist circumference; WHR: waist-to-hip ratio; BMI:

body-mass index; TC: total cholesterol; TG: triglyceride; HDL-C: high-density lipoprotein cholesterol; LDL-C: low-density lipoprotein cholesterol; HOMA: homeostatic model assessment;

IgE: immunoglobulin E.

*Binary logistic model. **Adjusted for age, sex, hypertension, BMI, TC, TG, hyperinsulinemia, hs-CRP, IgE, tryptase, and chymase.
